# Bread for the Aging Population: The Effect of a Functional Wheat–Lentil Bread on the Immune Function of Aged Mice

**DOI:** 10.3390/foods8100510

**Published:** 2019-10-18

**Authors:** Marina Carcea, Valeria Turfani, Valentina Narducci, Alessandra Durazzo, Alberto Finamore, Marianna Roselli, Rita Rami

**Affiliations:** Research Centre for Food and Nutrition, Council for Agricultural Research and Economics (CREA), via Ardeatina 546, 00178 Roma, Italy; valeria.turfani@crea.gov.it (V.T.); valentina.narducci@crea.gov.it (V.N.); alessandra.durazzo@crea.gov.it (A.D.); alberto.finamore@crea.gov.it (A.F.); marianna.roselli@crea.gov.it (M.R.); rita.rami@crea.gov.it (R.R.)

**Keywords:** wheat bread, lentil bread, bread composition, aged mice, immune function, intraepithelial lymphocytes, gut health

## Abstract

A functional bread tailored for the needs of the aging population was baked by substituting 24% of wheat flour with red lentil flour and compared with wheat bread. Its nutritional profile was assessed by analysing proteins, amino acids, lipids, soluble and insoluble dietary fibre, resistant starch, total polyphenols, lignans and the antioxidant capacity (FRAP assay). The wheat–lentil bread had 30% more proteins than wheat bread (8.3%, as is), a more balanced amino acids composition, an almost double mineral (0.63%, as is) as well as total dietary fibre content (4.6%, as is), double the amount of polyphenols (939.1 mg GAE/100g on dry matter, d.m.), higher amounts and variety of lignans, and more than double the antioxidant capacity (71.6 µmoL/g d.m.). The in vivo effect of 60 days bread consumption on the immune response was studied by means of a murine model of elderly mice. Serum cytokines and intraepithelial lymphocyte immunophenotype from the mice intestine were analysed as markers of systemic and intestinal inflammatory status, respectively. Analysis of immune parameters in intraepithelial lymphocytes showed significant differences among the two types of bread indicating a positive effect of the wheat–lentil bread on the intestinal immune system, whereas both breads induced a reduction in serum IL-10.

## 1. Introduction

According to recent statistics [1], the European population in particular is an aging one: In fact, the proportion of the population over 65 has steadily increased over the past decade. Aging is a condition that brings about a number of factors contributing to the risk of malnutrition which are related to physiological changes and medical and social conditions [2,3]. Current data for mean nutrient intakes suggest that, as a group, older adults are at risk of not meeting the recommended dietary allowance (RDA) or adequate intake (AI) values for calcium, vitamins, minerals, fibre [4] and protein [5]. It is well known that increased thresholds for taste and smell resulting in bland and uninteresting food tasting, coupled with impaired masticatory efficiency and swallowing difficulties can lead to consumption of a narrow, nutritionally imbalanced diet in the aged population [6,7,8]. Moreover, older adults have less money to spend on food.

All the above mentioned factors impact on the nutritional status of older adults, contributing to age-associated disorders including dysregulation of the immune system [9,10]. In fact, aging is associated with a declined immune function, a process known as immunosenescence, that negatively impacts on the capacity to properly respond to immune challenges thus contributing to the increased susceptibility of older persons to infections, poor vaccine efficacy and progressive development of low-grade, chronic inflammatory status [10,11,12]. 

Since a variety of bioactive dietary components have been shown to affect the immune system, an appropriate nutritional intervention may be a promising approach to counteract the impaired immune function occurring with aging [13,14]. Enriching staple or widely consumed foods can be a simple strategy to increase the intake of such components. Bread is an important food in the daily diet of several populations around the world. It is generally produced from refined white flour that lacks the nutrients, fibre and bioactive components present in the bran, but other ingredients can be added to increase the nutritional value of bread without altering its appearance and nature. 

Lentils have been gaining increasing interest in the development of healthy and functional foods, due to the fact of their nutritional properties [15,16,17,18,19,20]. The existing varieties of lentils vary in colour, size and texture, but they all have a low level of antinutrients and a mild taste [21]. Lentils contain 28.7%–31.5% protein, which is considerable among legumes, and provide the essential amino acids lysine and leucine [22]. They are a valuable source of dietary fibre, mainly the insoluble component, but also the soluble one [19]. Dietary fibres provide many health benefits, such as lowering serum levels of LDL cholesterol, glucose and blood pressure, reducing constipation and other intestinal disorders and preventing intestinal cancer [23]. Moreover, the soluble fibres of lentils contain nutritionally significant amounts of prebiotic molecules, such as galacto-oligosaccharides (GOS) and fructo-oligosaccharides (FOS), that are known to selectively stimulate the growth and/or activity of some beneficial bacteria in the colon, having the potential to improve host health, such as several *Bifidobacteria* and lactobacilli strains [24,25,26]. Finally, lentils are reported to have a high content of phenolic compounds and to show a high antioxidant activity [27]. Actually, phytochemicals, and among them phenolic compounds, are known to have a major impact on health, since they can provide therapeutic benefits including prevention and/or treatment of diseases and physiological disorders [28]. Amongst the lentil varieties, red lentils distinguish themselves for being an important source of proteins, fibre and particularly of bioactive substances [29,30].

A recent study from our laboratory showed that red lentil flour can be blended with wheat flour up to 24% to produce bread with good volume, pleasant texture and taste [31]. We thus engaged in further studies, which are reported in the present paper, to describe the nutritional profile of our 24% red lentil bread and to get some insight into the in vivo effect of its consumption, with particular regard to the aging condition. The bread nutritional profile was described by analysing proteins, amino acids, lipids, soluble and insoluble dietary fibre, resistant starch, total polyphenols and specifically lignans, which is an interesting group of polyphenols present in pulses; in addition, its antioxidant power was measured by the ferric reducing antioxidant power (FRAP) assay.

The same bread was chosen for an in vivo experiment with aged mice, used as a vulnerable animal model, to evaluate if a substitution of common wheat bread with this special wheat–legume bread could counteract the immune decline typical of older adults. The immune response was mainly assessed at the intestinal level, since the mucosal immune system, which is known to be also impaired in the older adults [32], represents the first line of contact with ingested antigens and molecules reaching the intestinal lumen. Some parameters, namely, serum cytokines and intraepithelial lymphocyte immunophenotype were analysed, as they represent markers of systemic and intestinal inflammatory status, respectively.

## 2. Materials and Methods

### 2.1. Flours and Bread Preparation

Commercial wheat flour (“0” type according to the Italian flour classification, Horeca brand) and commercial dehulled red lentils (Select, San Giuseppe Vesuviano, Napoli, Italy) were purchased from the market.

The wheat flour had a moisture level of 12.8% (International Association for Cereal Science and Technology (ICC) standard 110/1 [33]), ash 0.63% d.m. (indicated on the product label), total protein of 10.5% d.m. (product label), lipids of 0.8% d.m. (product label) and total dietary fibre of 3.2% d.m. of which 2.1% was insoluble and 1.1% soluble (measured according to Lee et al. [34] using a reagent kit (K-TDFR, Megazyme Int., Wicklow, Ireland)).

Red lentils were ground in a refrigerated laboratory mill (M20, Janke and Kunkel Ika Labortechnik, Staufen, Germany) (a cutting/impact mill with no sieve, operating at a speed of 20,000 rpm for 2 min) to produce a very homogeneous flour that had a moisture level of 10.3% (ICC standard 110/1 [33]), ash content of 2.39% dry matter (d.m.) (ICC standard 104/1 [33]), total protein of 24.6% d.m. (product label), lipids of 1.3% d.m. (product label) and total dietary fibre content of 17.1% d.m. of which 15.2% was insoluble and 1.9% soluble (measured according to Lee et al. [34] using a reagent kit (K-TDFR, Megazyme Int., Wicklow, Ireland)).

A blend was prepared by mixing wheat flour with red lentil flour in the proportions of 76% and 24%, respectively. These proportions were chosen according to the results of Turfani et al. [31], who determined the maximum amount of red lentil flour that could be added to wheat flour in order to avoid technical problems during bread making, such as excessive dough sticking, poor dough rheological properties and bread with unacceptably low volume, poor texture and excessive legume flavour.

The bread formulation was kept simple in order to study the nutritional properties of bread produced from the flour blend without additives. Loaves of bread were produced from wheat flour (wheat bread) and from a wheat–lentil flour blend by adapting the ICC standard method No. 131 [33] because solution 1 was not used, thus reducing sugar and eliminating ascorbic acid from the ingredients (the same adapted method was used in References [20,31]). Thus, 1000 g of flour blend were weighted at 14% m.b. and mixed with 15 g salt in the mixer bowl; the optimum water amount (previously determined by the Brabender Farinograph according to ICC Standard 151/1 [33]) was added to the flour blend, except for the small amount required to activate yeast; compressed baker’s yeast (18 g) was activated in 72 g of 5% sucrose solution (containing 68.4 g water and 3.6 g sucrose) at 35 °C for 10 min, then added to the flour blend. The dough was mixed for 10 min in a planetary bread mixer (Quick 20 by Sottoriva, Marano, Italy), then the dough temperature was checked (27 ± 1 °C) and the dough was fermented for 30 min in a fermentation cabinet at 30 °C with 85% relative humidity. After fermentation, the dough was scaled in four equal pieces, which were placed in baking tins and proofed for 50 min at 30 °C with 85% relative humidity, then baked for 30 ± 2 min at 220 °C in a convection/steam oven. The bread volume was determined within 20 ± 4 h by the rapeseed displacement method (AACCI Method 10-05.01) [35].

Bread for mouse feeding was baked all together at the beginning of the experiment to prevent variability due to the different preparation conditions, divided in aliquots sufficient for weekly diet preparation and frozen. Bread aliquots were thawed at room temperature at the moment of diet preparation.

### 2.2. Chemicals and Standards for Bread Analysis

The solvents used (i.e., acetone, diethyl ether, ethanol, ethyl acetate, methanol, n-hexane) were of HPLC or analytical grade and were purchased from Carlo Erba (Milan, Italy). Reagents were of the highest available purity. Hydrochloric acid 35%, formic acid 99%, glacial acetic acid, sulphuric acid 96%, tartaric acid, boric acid, sodium hydroxide, sodium hydroxide 32% solution, tris(hydroxymethyl)-aminomethane (TRIS), Folin–Ciocalteu reagent, sodium carbonate 20% solution, iron (II) sulphate heptahydrate (99%) and iron (III) chloride hexahydrate (97%–102%) were purchased from Carlo Erba. Kjieltabs (CuSO4/K2SO4), sulphuric acid solution 0.1 N and hydrogen peroxide 30% were purchased from VWR International PBI (Milan, Italy). Sodium citrate dihydrate, sodium acetate trihydrate, sodium chloride, glacial acetic acid, 2-metoxyethanol, ninhydrin were purchased from Merck-BDI (Darmstadt, Germany). Tin (II) chloride dehydrate, sodium acetate trihydrate 99%, MES (2(N-morpholino)-ethanesulpohonic acid), trolox (6-hydroxy-2,5,7,8-tetramethylchroman-2-carboxylic acid), TPTZ (2,4,6-Tris(2-pyridyl)-s-triazine) and Helix Pomatia µ-glucuronidase/sulphatase S9626–10KU Type H-1, 0.7 G solid, 14,200 units/g solid were purchased from Sigma–Aldrich (Milan, Italy). Standards were of the highest available grade: Amino acids standards and gallic acid monohydrate were purchased from Sigma–Aldrich, whereas isolariciresinol, secoisolariciresinol, lariciresinol and pinoresinol were from Chemical Research (Rome, Italy). Ultra-pure water was produced by using in sequence a Millipore Elix 5 system and a Millipore Synergy 185 system (Millipore, Molsheim, France).

### 2.3. Proximate Composition, Amino Acids, Total Polyphenols, Lignans and Antioxidant Properties of Bread

Moisture, proteins (conversion factor 6.25 for legume flours and 5.70 for wheat flour), lipids and ash were determined by standard ICC methods 110/1, 105/2, 136, 104/1, respectively [33]. Soluble (SDFs), insoluble (IDFs) and total (TDFs) dietary fibres were determined according to Lee et al. [32] using a reagent kit (K-TDFR, Megazyme Int., Wicklow, Ireland). Available carbohydrates were calculated by difference. Resistant starch was determined according to AACC Method 32-40.01 [31] by means of a reagent kit (RSTAR, Megazyme); however, the results of all determinations were below the limit of detection (2%) and they are not shown in the tables.

Amino acids were determined according to Spackman, Stein and Moore [36] using a Beckman System Gold 126 amino acid analyser (Beckman Coulter Inc., Brea, CA, USA) equipped with a Beckman Spherogel IEX High-Performance Sodium column P/N 727450 and a Beckman UV detector with ninhydrin reactor. The samples were hydrolysed in hydrochloric acid 6 M under vacuum in sealed tubes at 105 °C for 24 h. For the determination of valine and isoleucine, the hydrolysis lasted 72 h. For cysteine and methionine, the samples were oxidised by oxygen peroxide and formic acid (88%) at 0 °C for 4 h at first, then the reagents were removed by evaporation under vacuum and the residue was hydrolysed in hydrochloric acid 6 M at 105 °C for 22 h. After hydrolysis and removal of the excess HCl, residues were re-dissolved in citrate buffer 0.2 M and injected.

Total polyphenols (TPCs) were extracted from samples as described by Durazzo et al. [30] in two separate fractions. Free polyphenols were extracted in methanol/water 1:1 and acetone/water 3:7. The residue was treated with hot sulphuric acid in methanol in order to free the hydrolysable polyphenols. The polyphenol content in the aqueous–organic extract and in the hydrolysed residue was determined by means of the Folin–Ciocalteau reagent [37], by measuring absorbance at 760 nm and using gallic acid as a standard.

For the analysis of lignans, samples were preliminarily defatted with hexane and diethyl ether for 8 h in a Soxhlet apparatus. The lignans were extracted and analysed by High Performance Liquid Chromatography (HPLC) as in Durazzo et al. [30]. The HPLC analyses were performed with a 50 µL extract using an ESA-HPLC system (ESA, Chelmsford, MA, USA) consisting of an ESA Model 540 autoinjector, an ESA Model 580 solvent delivery module with two pumps, an ESA 5600 eight channels coulometric electrode array detector and the ESA CoulArray operating software which controlled all the equipment and carried out data processing. A SUPELCOSIL LC-18 column (25 cm × 4.6 mm, 5 µm) with a Perisorb Supelguard LC-18 (Supelco, Milan, Italy) was used. Isolariciresinol, lariciresinol, secoisolariciresinol and pinoresinol were detected and quantified.

The antioxidant properties were determined by means of the FRAP assay according to Durazzo et al. [30].

### 2.4. In Vivo Experiments: Experimental Design, Animals and Diets

The Balb/c aged mice (20 months old) were kept at 23 °C with a 12 h light–dark cycle and fed ad libitum with standard laboratory diets. Mice had free access to food and water. Body weight and food intake were recorded every week and every other day, respectively. After one week of adaptation, animals were randomly divided into three groups (6 animals per group), receiving three different diets for two months (60 days): One group was fed a standard control diet (control group, 20% casein, Laboratorio Dottori Piccioni, Gessate, Milan, Italy), one group was fed the wheat bread containing diet (wheat bread group), and a third group was fed the wheat–lentil bread containing diet (wheat–lentil bread group). The standard control diet was prepared using as reference the AIN-93M formulation [38]. The bread containing diets were appropriately balanced and were isocaloric in respect to the control diet. At the end of the experimental periods, animals were fasted for 16 h, anesthetised with intraperitoneal injection of pentobarbital (10 mg/kg) and sacrificed. Blood was drawn via cardiac puncture, whereas small intestine and colon were excised and immediately placed in cold phosphate buffered saline (PBS). The animal experiments were carried out in strict accordance with the recommendation of the European Guidelines for the Care and Use of Animals for Research Purposes. All experimental procedures complied with the Animal Care and Use Committee of the CREA—Research Centre for Food and Nutrition—and were approved by the National Health Ministry, General Direction of Animal Health and Veterinary Drugs (agreement number 0006828/03/02/2014). All efforts were made to minimise the suffering of the animals.

### 2.5. Intraepithelial Lymphocytes (IELs) Preparation

The intraepithelial lymphocytes (IELs) were prepared from jejunum and colon. Briefly, intestines were placed on ice in 10 mL RPMI-1640 medium (Sigma–Aldrich, Milan, Italy), washed twice with cold PBS, longitudinally opened and cut into small size pieces. Intestinal pieces were washed in Hank’s balanced salt solution (HBSS) and stirred twice for 45 min at 37 °C in an orbital shaker in HBSS added with 100 g/L foetal calf serum (FCS, Euroclone, Milan, Italy), 1 × 105 U/L penicillin, 100 mg/L streptomycin, 1 mM ethylendiamin-tetraacetic acid (EDTA), 5 mM Hepes, 1 mM dithiothreitol. The solution was passed through 100 and 40 μm nylon cell strainers (BD Falcon, Milan, Italy) and centrifuged at 650× *g*. The IELs were isolated from enterocytes by discontinuous 440/670 g/L Percoll gradient (PercollTM, GE Healthcare, Milan, Italy) in RPMI-1640 medium, and centrifuged at 650× *g* for 25 min.

### 2.6. Flow Cytometry Analysis of IELs Subpopulations

The following monoclonal antibodies were used for lymphocyte surface staining: Fluorescein isothiocyanate (FITC) anti-CD3 (clone 17.12), phycoerythrin (PE) anti-CD4 (clone GK1.5), phycoerythrin–cyanine 5 (PE-Cy5) anti-CD8 (clone 53-67), PE anti-CD19 (clone ID3), peridinin–chlorophyll-protein (PerCP) anti-CD45 (clone 30-F11), PE anti-TCR γδ (clone GL3), PE-Cy5 anti-TCR αβ (clone H57-597) and anti-CD16/CD32 (clone 2.4G2) (BD Pharmingen, Milan, Italy). Each antibody was previously titrated to determine the optimal concentration for maximal staining. The IELs (1 × 106 cells) were pre-incubated for 20 min with anti-CD16/CD32 to block Fc receptors. Cells were then washed and labelled with the appropriate mixture of antibodies for 30 min, centrifuged at 650× *g* and resuspended in FacsFlow (BD Biosciences, Milan, Italy). Flow cytometry analysis was performed using a FACSCalibur instrument (BD Biosciences). To exclude dead/dying cells and, therefore, non-specific antibody-binding cells, lymphocytes were gated according to forward and side scatter. The percentage of B and T lymphocytes was calculated on leukocyte (CD45+) gate, whereas the CD4+, CD8+ and CD4+CD8+ subsets, as well as αβ and γδ lymphocytes, were calculated on T lymphocyte (CD3+) gate. At least 10,000 events were acquired. Data were analysed using CellQuest software (BD Biosciences).

### 2.7. Analysis of Inflammatory Status in Mice Intestine

Small parts of jejunum and colon (1 cm) were immediately washed in cold PBS to remove stools and frozen in liquid nitrogen. To evaluate the inflammatory status of intestine, frozen tissues were weighted and homogenised in cold radioimmunoprotein (RIPA: 20 mM Tris-HCl pH 7.5, 150 mM NaCl, 0.1% Sodium Dodecyl Sulfate (SDS), 1% Na deoxycholate, 1% Triton X- 100) assay buffer supplemented with 1 mM phenylmethylsulphonyl fluoride, protease inhibitor cocktail (Complete Mini, Roche, Milan, Italy) and phosphatase inhibitor cocktail (PhosSTOP, Roche). Protein concentration was measured by the Lowry assay. Intestinal homogenates (50 µg total proteins) were dissolved in sample buffer (50 mM Tris-HCl, pH 6.8, 2% SDS, 10% glycerol, 100 g/L bromophenol blue, 10 mM beta-mercaptoethanol), heated for 5 min, fractionated by SDS-polyacrylamide gel (4–20% gradient) electrophoresis and transferred to 0.2 μm nitrocellulose filters (Trans-Blot Turbo, Biorad, Milan, Italy). Membranes were incubated with the following primary antibodies: Rabbit polyclonal anti-mouse NF-kB p65 and P-p65. Proteins were detected with horseradish peroxidase conjugated secondary antibodies (Cell Signaling Technology, Danvers, MA) and enhanced chemiluminescence reagent (ECL kit LiteAblot Extend, Euroclone, Milan, Italy), followed by analysis of chemiluminescence with the charge-coupled device camera detection system Las4000 Image Quant (GE Health Care Europe GmbH, Milan, Italy). The expression of the P-p65 proteins was normalised to their corresponding unphosphorylated forms.

### 2.8. Cytokine Secretion in Mice Serum

Blood samples were collected in test tubes, centrifuged (2000× *g* for 10 min at 4 °C) and the supernatant (serum) was stored at −80 °C until further analysis. The levels of cytokines and chemokines were analysed using Bio-plex/Luminex technology (mouse magnetic Luminex screening assay, Labospace, Milan, Italy). Briefly, Luminex multi-analyte profiling is a multiplexing technology allowing simultaneous analysis of up to 500 bioassays from a small sample volume. The following cytokines and chemokines were simultaneously detected in 50 µL undiluted samples: Tumour necrosis factor (TNF)-alpha, granulocyte macrophage-colony stimulating factor (GM-CSF), regulated upon activation normal T cell expressed and secreted (RANTES), interleukin (IL)-23, IL-17, IL-10, IL-12 and IL-6.

### 2.9. Presentation of Results and Statistics

Proximate composition, dietary fibre and lignan analyses were performed in triplicate whereas four replicates were used for polyphenols and FRAP. Mean values and percent coefficient of variation (%CV) are reported, together with the significance level of Student’s *t*-test between wheat bread and wheat–lentil bread. Amino acids were analysed by a single determination without replicates. Calculations were performed by means of Microsoft Excel and PAST statistical package, version 2.17c [39].

For the results of in vivo experiments, values in graphs and tables represent means and %CV. Statistical significance was evaluated by one-way ANOVA followed by post-hoc Tukey’s honestly significant difference (HSD) test. Normal distribution and homogeneity of variance of data were previously verified with appropriate statistical tests. Differences with *p*-values < 0.05 were considered significant. Statistical analysis was performed with the PAST statistical package.

## 3. Results

Table 1 shows the proximate composition of wheat bread and of the wheat–lentil bread. The two breads did not significantly differ in their moisture content (38.9% as is basis and 40.0% as is basis for the wheat bread and the wheat–lentil bread, respectively), whereas significant differences were observed for protein (6.4% as is basis and 8.3% as is basis for the wheat and the wheat–lentil bread, respectively), ash (0.39% as is basis and 0.63% as is basis for the wheat and the wheat-lentil bread, respectively) and IDF (1.6% as is basis and 3.1% as is basis for the wheat and the wheat-lentil bread, respectively).

The content of 17 amino acids in mg/100 g proteins (eight essentials, tryptophan was not determined, plus alanine, arginine, aspartic acid, cysteine, glutamic acid, glycine, proline, serine and tyrosine) in both wheat and wheat–lentil bread is reported in Table 2. The main differences observed were aspartic acid (4.20 and 6.05 for the wheat and the wheat–lentil bread, respectively), glutamic acid (39.75 and 34.36 for the wheat and the wheat–lentil bread, respectively), proline (9.90 and 8.39 for the wheat and the wheat–lentil bread, respectively), lysine (2.18 and 3.30 for the wheat and the wheat–lentil bread, respectively) and arginine (3.89% and 4.91% for the wheat and the wheat–lentil bread, respectively).

Data on total polyphenols content (TPC) (both in the aqueous organic extract and in the hydrolysable residue), four lignans content—namely, isolariciresinol, lariciresinol, secoisolariciresinol, pinoresinol—and the antioxidant power measured by the FRAP (both in the aqueous organic extract and hydrolysable residue) in our experimental wheat and wheat–lentil bread are reported in Table 3.

With regards to TPC, significant differences were observed between the wheat bread and the wheat–lentil bread both in the aqueous organic extract and the hydrolysable residue with values of 59.4 and 250.0 mg GAE/100 g d.m. in the aqueous organic extract of wheat and wheat–lentil bread, respectively, and higher values of 411.8 and 689.1 in the hydrolysable residue of the same samples.

With regards to the content of the four determined lignans, lariciresinol and pinoresinol were not detectable in the wheat bread whereas they reached 45.2. and 27.3 µg/100g d.m., respectively, in wheat–lentil bread. Significant differences between the two types of bread were observed for isolariciresinol (2.4 and 66.5 µg/100g d.m. for wheat and wheat–lentil bread, respectively) and for secoisolariciresinol (4.5 and 7.0 µg/100 g d.m. for wheat and wheat–lentil bread, respectively). Significant differences were also observed in the FRAP values of the aqueous organic extract and the hydrolysable residue of both types of bread which were higher for lentil bread in both cases (21.9 versus 6.4 and 49.7 versus 21.1 µmoL/g d.m., respectively).

Data on the composition of the diets which were given to the aged mice are reported in Table 4.

Table 5 reports the data relative to mice initial (i.e., at the beginning of treatment) and final (i.e., at the end of treatment) body weight, as well as daily food intake. No significant differences were observed among the three groups in body weight nor in food intake.

Among all the analysed cytokines and chemokines in serum, only three resulted at detectable levels: The anti-inflammatory IL-10, the pro-inflammatory IL-17 and the GM-CSF chemokine. Interleukin-10 significantly decreased in the wheat and wheat–lentil bread-treated animals as compared to control, whereas no significant differences were observed in IL-17 and GM-CSF levels among the three groups (Table 6).

**Figure 1:**
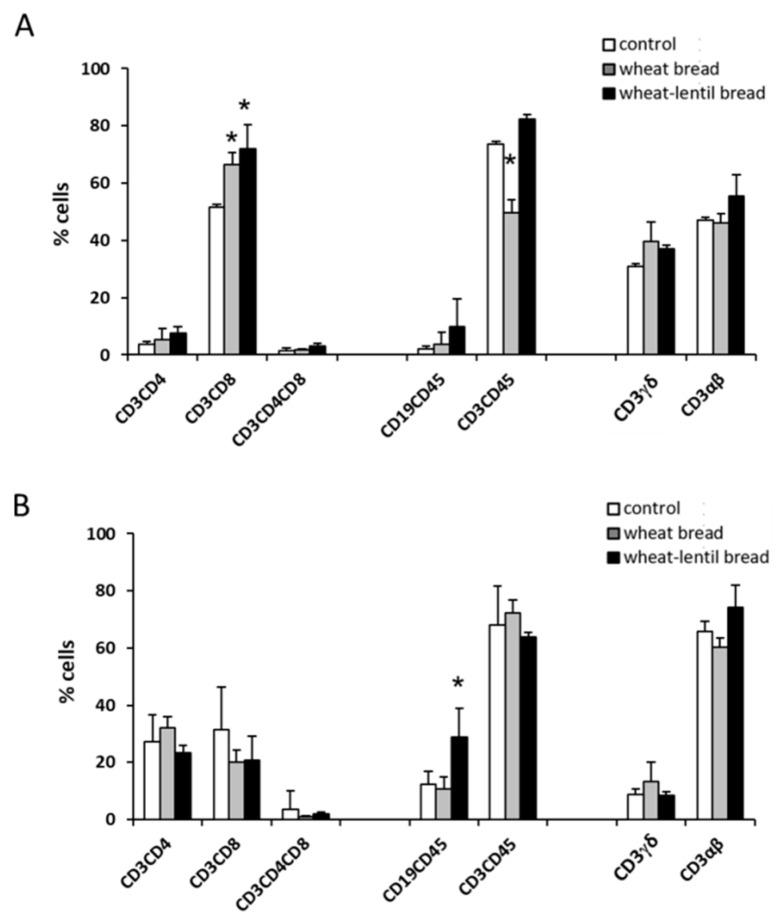
Intraepithelial lymphocyte (IEL) subpopulations in the jejunum (**A**) and colon (**B**) of mice fed a control, a wheat bread and a wheat–lentil diet measured by flow cytometry. (The percentage of B and T lymphocytes was calculated on leukocyte (CD45+) gate, whereas the CD4+, CD8+ and CD4+CD8+ subsets, as well as αβ and γδ lymphocytes, were calculated on T lymphocyte (CD3+) gate). Data represent the means ± SD of 6 mice. * *p* < 0.05 versus control.

The IELs subpopulation percentages in jejunum (panel A) and colon (panel B) of mice fed control, wheat bread or wheat–lentil bread diets are presented in Figure 1. Histograms show a significant increase of cytotoxic T cell (CD3+CD8+) percentages in the jejunum of mice fed both types of bread compared to the control diet, whereas the percentage of total T cells (CD3+CD45+) were reduced in mice fed wheat bread compared to control and wheat–lentil bread. In the colon, only a significant increase of B cell (CD19+CD45+) percentages was observed in mice fed wheat–lentil bread. No differences in the percentages of other lymphocyte subpopulations were observed among the groups.

Western blot analysis of the phosphorylated form of the p65 subunit of NF-kB in the jejunum and colon of mice did not show any significant difference among groups, indicating that the treatment with wheat and wheat–lentil bread did not induce an inflammatory status in the mice intestine (data not shown).

## 4. Discussion

As expected, the proximate composition of the wheat and the wheat–lentil bread mirrored the proximate composition of the flours of origin (see the Materials and Methods section and Table 1). In fact, the wheat–lentil bread contained 30% more proteins than wheat bread, it had an almost double ash content, therefore a higher level of minerals in general, together with an almost double amount of total dietary fibre, especially the insoluble component. Moreover, the lentil–wheat bread contained a lower amount of available carbohydrates than wheat bread.

Besides having a higher protein content, wheat–lentil bread had a more balanced amino acid profile than wheat bread (Table 2). Indeed, the amino acid profiles of wheat and lentils are complementary. For example, lysine is abundant in lentils, whereas sulphur amino acids are present in higher amounts in wheat. Lentil proteins are, in fact, mainly constituted by globulins and albumins [40] and, thus, have a different composition from wheat proteins, which are mainly constituted of prolamins and glutelins. The presence of the lentil flour increases the level of almost all the essential amino acids in bread (Table 2).

The lower amount of available carbohydrates in wheat–lentil bread is due to the fact that lentils contain less starch than wheat (about 40–45%, [18]); it is also reported that legume starch has a higher fraction of amylose than wheat (about 35%, [41]).

Regarding dietary fibre, both wheat and wheat–lentil breads contain only a small amount of the soluble component, around 1%; however, the soluble fibre of lentils is reported to contain beta glucans [18]. Βeta-glucans are very interesting from a functional point of view, because they are known to induce a variety of physiological effects with a positive impact on health, acting in particular through immunomodulatory pathways, that can suppress cancer proliferation, lower cholesterol levels and thus reduce the risk for cardiovascular disease [42,43].

The wheat–lentil bread was richer in phenolic substances, in particular those present in the aqueous organic extract, than wheat bread and this is the reason why it also had better antioxidant properties (Table 3). The soluble free phenolics found in the extract come mainly from cellular vacuoles whereas the insoluble phenolics present in the residue are bound to other components mainly fibre. The hydrolysable bound phenols represent the main polyphenol fraction in both bread types (between 73% and 87% of TPC). The literature reports that significant amounts of phenolic compounds remain in the extraction residues, associated with the food matrix [44]. The phenolic molecules most frequently found in cereals are phenolic acids and flavonoids whereas in pulses we also find tannins [45]. Polyphenols in general, both the free and the bound ones, thanks to their antioxidant properties are considered to exert a protective effect on human health [46].

The lignans, secoisolariciresinol and isolariciresinol, were found in both breads (Table 3). However, the wheat–lentil bread not only contained higher amounts of these lignans, but also had additional lignan types and, in particular, lariciresinol and pinoresinol in the following order: Isolariciresinol > lariciresinol > pinoresinol > secoisolariciresinol. These results are in agreement with data on lignan content in legume flours reported by Durazzo et al. [30]. Literature data indicate flaxseed and sesame as major alimentary sources of lignans and rye and lentils as good sources [30,47]. Lignans are a large group of polyphenols of increasing interest because their intake has been related to beneficial health effects, including cancer and cardiovascular disease prevention [48]. In this regard it is interesting to report that in 2012 the research group of During et al. [49] published a paper to report on their investigation of whether plant lignans are taken up by intestinal cells and modulate the intestinal inflammatory response using the Caco-2 cell model. Their findings suggest that plant lignans can be absorbed and metabolised in the small intestine and, among the plants lignans tested, pinoresinol exhibited the strongest anti-inflammatory properties.

The antioxidant power as measured by FRAP was significantly higher in wheat–lentil bread than in wheat bread. The FRAP assay is a quick and sensitive way to measure the antioxidant capacity of biological samples. In both cases, the hydrolysable residue had a higher FRAP value than the aqueous–organic extract thus providing the major contribution to the total antioxidant power (from 69% to 77%); this matches the results of the total polyphenols content. Thus, a bread recipe where about one-quarter of the wheat flour is substituted by red lentil flour more than doubles the antioxidant capacity of bread (Table 3).

Concerning animal experiments, our first consideration was that no significant differences were observed in body weight and food intake among the groups of mice fed the control and the two types of bread diets; this indicates that the different diets had the same palatability for the mice, and that they did not impact on eating behaviour and appetite; and that all the differences observed in our sets of data were due to differences in the composition of the diets.

Analysis of the immune parameters in IELs isolated from jejunum showed an increase of cytotoxic T lymphocytes (CD3+CD8+) percentages in both wheat and wheat–lentil bread-treated animals, as compared to the control. Moreover, total T lymphocytes (CD3+CD45+) were significantly reduced in the wheat bread group and increased in the wheat–lentil bread group, compared to the control (Figure 1A). The IEL subpopulation’s analysis in colon showed a significant increase of the B lymphocytes (CD19+CD45+) percentage in lentil bread-treated animals as compared to wheat bread and the control (Figure 1B). In this regard, we could hypothesise a role of the higher amount of β-glucans in the lentil bread while not ignoring that such compounds can increase the percentage of activated B lymphocytes and stimulate immune response [50,51].

We can say that the results of our study indicate a positive effect of wheat–lentil bread supplementation on the intestinal immune system of aged mice, as this supplementation was able to counteract some of the immune alterations typical of the older adults. In fact, aging is characterised by intrinsic changes in hematopoietic precursors that affect their proliferative potential, and this represents a key factor contributing to age-related decline in B- and T-cell production [52]. Thus, the increase of total T lymphocytes indicates a better immune response, and the increase of cytotoxic T lymphocytes suggests an improved capacity to respond to toxic agents and/or pathogens, that is known to be reduced in older adults. We can also hypothesise that the increase of B lymphocytes in the colon indicates a more efficient antibody response. In fact, it is well known that the antibody response is impaired in the older adults [53]. Moreover, it has been largely demonstrated that an antioxidants-containing diet may ameliorate lymphocyte response and protect immune cells from oxidative stress-induced apoptosis [54]. Besides polyphenols in general, the positive effects on the immune system in our specific case could also be ascribed to the significantly higher amount of the lignan isolariciresinol (27 times higher) and the presence of the lignan pinoresinol in wheat–lentil bread compared to wheat bread; these two lignans in particular have been shown to exert immunomodulatory and anti-inflammatory effects [49,55].

No significant differences were observed in the other analysed IELs subpopulations (Figure 1A,B).

Among all the analysed cytokines in serum, only IL-10 was significantly decreased in the wheat and wheat–lentil bread treated animals as compared to the control (Table 6). The role of IL-10 in older adults is controversial; while some studies report that IL-10 increased the inflammatory status, others indicate that this cytokine plays a key role as an anti-inflammatory factor [56,57]. It has also been reported that aging is associated with an increase of IL-10 that, together with other cytokines, could be considered as a marker of frailty [58,59].

## 5. Conclusions

It is increasingly coming to general attention that the aging population needs to eat appropriately to prevent and reduce all the health risks associated with this phase of human life. In other words, there is a need for tailored foods for aging people. Enriching staple or widely consumed foods can be a simple strategy to guaranty the intake of key nutrients able to have a beneficial effect on the negative aspects associated with aging such as the decline of the immune function. Based on previous studies done in our laboratory, we identified bread as a target food and red lentil flour as a raw material useful to add functionality to bread. We also identified technological constraints that allowed a maximum addition of 24% lentil flour.

For the purpose of this study, we baked two kinds of bread: A common wheat bread and a wheat–24% lentil flour bread. The chemical analysis of the bread components showed that the wheat–lentil bread had 30% more proteins than wheat bread coupled with a more balanced amino acid composition; it had an almost double mineral as well as total dietary fibre content, especially the insoluble component, double the amount of polyphenols, an interesting lignans content and more than double the antioxidant capacity. Thus, this wheat–lentil bread proved to be nutritionally richer and more functional than common wheat bread.

The in vivo effect of the consumption of wheat–lentil bread versus wheat bread on the immune response was studied by means of a murine model of aged mice. Analysis of the immune parameters in intraepithelial lymphocytes isolated from the mice intestine showed significant differences between the two types of bread indicating a positive effect of the lentil–wheat bread on the intestinal immune system. Cytokines in serum were also analysed. Considering that IL-10 is indicated as a frailty marker, we suppose that wheat and wheat–lentil breads in diets could have a positive effect on inflammatory status and improve the health status of aged mice.

This study clearly demonstrates that this is possible by substituting wheat flour with another suitable flour to manufacture a simple and well-accepted food, such as bread, which shows more functionality and is more tailored for the aging population than traditional, common bread with soft wheat only.

## Figures and Tables

**Table 1:** Proximate composition of wheat and wheat–lentil bread (76% wheat flour/24% red lentil) ^#^.

Bread	Moisture%		Protein%		Fat%		Ash%		IDF ^§^%		SDF ^§^%		TDF ^§^%		Available Carbohydrates (by Difference)%	
	Mean ^ns^	CV	Mean **	CV	Mean ^ns^	CV	Mean **	CV	Mean *	CV	Mean ^ns^	CV	Mean ^ns^	CV	Mean ^ns^	CV
Wheat bread	38.9	0.8%	6.4	1.6%	1.0	0%	0.39	0%	1.6	13%	1.0	20%	2.6	15%	50.8	1.6%
Wheat–lentil bread	40.0	0.8%	8.3	1.2%	0.9	3%	0.63	0.02%	3.1	16%	1.5	60%	4.6	28%	45.5	4.0%

^#^ Values are the mean of three replicates, on wet basis; ^§^ IDF—insoluble dietary fibre; SDF—soluble dietary fibre; TDF—total dietary fibre; *, **, ^ns^ significance (*t*-test) of difference among the two samples: * significant at *p* < 0.05; significant ** at *p* < 0.01; ^ns^ not significant.

**Table 2:** Amino acid composition of wheat and wheat–lentil bread (mg/100 g proteins) ^#, §^.

**Sample**	**Aspartic Acid**	**Threonine**	**Serine**	**Glutamic Acid**	**Proline**	**Glycine**	**Alanine**	**Cystine**	**Valine**
Wheat bread	4.20	2.82	4.98	39.75	9.90	3.67	3.03	1.89	4.53
Wheat–lentil bread	6.05	3.01	5.04	34.36	8.39	3.79	3.27	1.60	4.84
**Sample**	**Methionine**	**Isoleucine**	**Leucine**	**Tyrosine**	**Phenylalanine**	**Histidine**	**Lysine**	**Arginine**	**NH_4_**
Wheat bread	1.36	4.07	7.08	2.77	4.88	2.28	2.18	3.89	5.12
Wheat–lentil bread	1.13	4.27	7.21	2.76	4.92	2.41	3.30	4.91	4.42

^#^ Amino acids were analysed as a single determination without replicates. ^§^ Tryptophan was not analysed.

**Table 3:** Total polyphenols, lignans and FRAP of wheat and wheat–lentil bread ^#^.

Bread	TPC (mg GAE/100 g d.m.)				Lignans (µg/100 g d.m.)								FRAP (µmol/g d.m.)			
	Aqueous–Organic Extract		Hydrolysable Residue		Isolariciresinol		Lariciresinol		Secoisolariciresinol		Pinoresinol		Aqueous–Organic Extract		Hydrolysable Residue	
	Mean **	CV	Mean **	CV	Mean *	CV	Mean	CV	Mean **	CV	Mean	CV	Mean **	CV	Mean **	CV
Wheat bread	59.4	1.5%	411.8	0.3%	2.4	8.3%	n.d.		4.5	4.4%	n.d.		6.4	3.1%	21.1	12.3%
Wheat–lentil bread	250.0	2.0%	689.1	4.9%	66.5	18.6%	45.2	12.2%	7.0	4.3%	27.3	18%	21.9	12.8%	49.7	7.8%

^#^ Values are means of four to seven replicates; GAE—gallic acid equivalent; d.m.—dry matter; TPC—total phenols content; FRAP—ferric reducing antioxidant power; n.d.—not detectable. *, ** Significance (*t*-test) of differences among the two samples: Significant ** at *p* < 0.01 and * at *p* < 0.05.

**Table 4:** Diets composition.

Component	Control (g/kg)	Wheat Bread (g/kg)	Wheat–Lentil Bread (g/kg)
Bread		465.7	465.7
Maize starch	465.7	66.4	92.6
Casein	140.0	88.8	74.8
Maltodextrins	155.0	155.0	155.0
Sucrose	100.0	100.0	100.0
Soya oil	40.0	35.6	35.4
Cellulose	50.0	39.2	27.2
Saline mix	35.0	35.0	35.0
Vitamin mix	10.0	10.0	10.0
L-cystine	1.8	1.8	1.8
Choline chloride	2.5	2.5	2.5
TBHQ ^#^	0.008	0.008	0.008

^#^ tert-Butylhydroquinone.

**Table 5:** Body weight and daily food intake of control, wheat bread and wheat–lentil bread fed mice *.

Diet	Initial Body Weight (g)		Final Body Weight (g)		Food Intake (g/day)	
	Mean	CV	Mean	CV	Mean	CV
Control	24.0	8.3%	25.0	9.6%	3.6	22.2%
Wheat bread	25.5	8.2%	25.5	12.9%	3.4	23.5%
Wheat–lentil bread	24.0	15.0%	25.7	5.8%	3.7	24.3%

* Data represent means and %CV of 6 mice per group.

**Table 6:** Cytokine serum secretion ^#^.

Diet	Cytokine (pg/mL)					
	IL-17		IL-10		GM-CSF	
	Mean	CV	Mean	CV	Mean	CV
Control	25.79	4.96%	13.49	55.75%	3.02	10.26%
Wheat bread	23.01	10.30%	4.81 *	64.66%	2.50	22.40%
Wheat–lentil bread	23.52	1.0%	6.73 *	26.60%	2.65	3.77%

^#^ Data represent means and %CV of 6 mice per group; * *p* < 0.05 versus control.

## References

[1] EUROSTAT (2019). Your Key to European Statistics. https://ec.europa.eu/eurostat/data/database.

[2] Morley J.E. (2012). Undernutrition in older adults. Fam. Pract..

[3] Starr K.N.P., McDonald S.R., Bales C.W. (2015). Nutritional vulnerability in older adults: A continuum of concerns. Curr. Nutr. Rep..

[4] Lichtenstein A.H., Rasmussen H., Yu W.W., Epstein S.R., Russell R.M. (2008). Modified MyPyramid for older adults. J. Nutr..

[5] Ziylan C., Haveman-Nies A., Kremer S., de Groot L.C. (2016). Protein-enriched bread and readymade meals increase community-dwelling older adults’ protein intake in a double-blind randomized controlled trial. J. Am. Med. Dir. Assoc..

[6] Boyce J.M., Shone G.R. (2006). Effects of ageing on smell and taste. Postgrad. Med. J..

[7] Ney D.M., Weiss J.M., Kind A.J., Robbins J. (2009). Senescent swallowing: Impact, strategies, and interventions. Nutr. Clin. Pract..

[8] Rémond D., Shahar D.R., Gille D., Pinto P., Kachal J., Peyron M.A., Dos Santos C.N., Walther B., Bordoni A., Dupont D. (2015). Understanding the gastrointestinal tract of the elderly to develop dietary solutions that prevent malnutrition. Oncotarget.

[9] Chandra R.K. (2002). Nutrition and the immune system from birth to old age. Eur. J. Clin. Nutr..

[10] Fülöp T., Dupuis G., Witkowski J.M., Larbi A. (2016). The role of immunosenescence in the development of age-related diseases. Rev. Investig. Clín..

[11] Castelo-Branco C., Soveral I. (2014). The immune system and aging: A review. Gynecol. Endocrinol..

[12] Pera A., Campos C., López N., Hassouneh F., Alonso C., Tarazona R., Solana R. (2015). Immunosenescence: Implications for response to infection and vaccination in older people. Maturitas.

[13] Pae M., Meydani S.N., Wu D. (2012). The Role of nutrition in enhancing immunity in aging. Aging Dis..

[14] Vulevic J., Juric A., Walton G.E., Claus S.P., Tzortzis G., Toward R.E., Gibson G.R. (2015). Influence of galacto-oligosaccharide mixture (B-GOS) on gut microbiota, immune parameters and metabonomics in elderly persons. Br. J. Nutr..

[15] Dalgetty D.D., Baik B.K. (2006). Fortification of bread with hulls and cotyledon fibers isolated from peas, lentils and chickpeas. Cereal Chem..

[16] Borsuk Y., Arntfield S., Lukow O.M., Swallow K., Malcolmson L. (2012). Incorporation of pulse flours of different particle size in relation to pita bread quality. J. Sci. Food Agric..

[17] Baik B.K., Han I.H. (2012). Cooking, roasting and fermentation of chickpeas, lentils, peas and soybeans for fortification of leavened bread. Cereal Chem..

[18] Faris M.A.I.E., Takruri H.R., Issa A.Y. (2012). Role of lentils (Lens culinaris L.) in human health and nutrition: A review. Med. J. Nutr. Metab..

[19] Aslani Z., Alipour B., Mirmiran P., Bahadoran Z. (2015). Lentil’s (Lens culinaris L.) functional properties in prevention and treatment of non-communicable chronic diseases: A review. Int. J. Nutr. Food Sci..

[20] Turfani V., Narducci V., Durazzo A., Galli V., Carcea M. (2017). Technological, nutritional and functional properties of wheat bread enriched with lentil or carob flours. LWT-Food Sci. Technol..

[21] Nienke M., Lindeboom T., Baga M., Vandenberg A., Chibbar R.N. (2011). Composition and correlation between major seed constituents in selected lentil (Lens culinaris. Medik) genotypes. Can. J. Plant Sci..

[22] Roy F., Boye J.I., Simpson B.K. (2010). Bioactive proteins and peptides in pulse crops: Pea, chickpea and lentil. Food Res. Int..

[23] Hefnawy T.H. (2011). Effect of processing methods on nutritional composition and anti-nutritional factors in lentils (Lens culinaris). Ann. Agric. Sci..

[24] Johnson C.R., Thavarajah D., Combs G.F., Thavarajah P. (2013). Lentil (*Lens culinaris* L.): A prebiotic-rich whole food legume. Food Res. Int..

[25] Gibson G.R., Hutkins R., Sanders M.E., Prescott S.L., Reimer R.A., Salminen S.J., Scott K., Stanton C., Swanson K.S., Cani P.D. (2017). Expert consensus document: The International Scientific Association for Probiotics and Prebiotic s (ISAPP) consensus statement on the definition and scope of prebiotics. Nat. Rev. Gastroenterol. Hepatol..

[26] Micioni Di Bonaventura M.V., Cecchini C., Vila-Donat P., Caprioli G., Cifani C., Coman M.M., Cresci A., Fiorini D., Ricciutelli M., Silvi S. (2017). Evaluation of the hypocholesterolemic effect and prebiotic activity of a lentil (*Lens culinaris* Medik) extract. Mol. Nutr. Food Res..

[27] Johari A., Arora S., Potaliya M., Kawatra A. (2015). Role of Lentil (*Lens culinaris* L.) in human health and nutrition. Ann. Agri-Bio Res..

[28] Bouchenak M., Lamri-Senhadji M. (2013). Nutritional quality of legumes, and their role in cardiometabolic risk prevention: A review. J. Med. Food.

[29] Wang N., Hatcher D.W., Toews R., Gawalko E.J. (2009). Influence of cooking and dehulling on nutritional composition of several varieties of lentils (Lens culinaris). Food Sci. Technol..

[30] Durazzo A., Turfani V., Azzini E., Maiani G., Carcea M. (2013). Phenols, lignans and antioxidant properties of legume and sweet chestnut flours. Food Chem..

[31] Turfani V., Narducci V., Mellara F., Bartoli L., Carcea M. (2017). Technological properties and bread characteristics of soft wheat and red lentil flour blends. Tec. Molit. Int..

[32] Mabbott N.A., Kobayashi A., Sehgal A., Bradford B.M., Pattison M., Donaldson D.S. (2015). Aging and the mucosal immune system in the intestine. Biogerontology.

[33] International Association for Cereal Science and Technology (2003). ICC Standard Methods.

[34] Lee S.C., Prosky L., DeVries J.W. (1992). Determination of total, soluble and insoluble, dietary fibre in foods, enzymatic-gravimetric method, MES-TRIS buffer: Collaborative study. J. Assoc. Off. Anal. Chem..

[35] AACC (2009). International Approved Methods of Analysis.

[36] Spackman D.K., Stein W.H., Moore S. (1958). Chromatography of amino acids on sulfonated polystyrene resin: An improved system. Anal. Chem..

[37] Singleton V.L., Orthofer R., Lamuela-Raventós R.M. (1999). Analysis of total phenols and other oxidation substrates and antioxidants by means of Folin-Ciocalteu reagent. Methods Enzymol..

[38] Reeves P.G. (1997). Components of the AIN-93 diets as improvements in the AIN-76A diet. J. Nutr..

[39] Hammer Ø., Harper D.A.T., Ryan P.D. (2001). PAST: Paleontological Statistics Software Package for Education and Data Analysis. Palaeontol. Electron..

[40] Boye J., Zare F., Pletch A. (2010). Pulse proteins: Processing, characterization, functional properties and applications in food and feed. Food Res. Int..

[41] Joshi M., Aldred P., Panozzo J.F., Kasapis S., Adhikari B. (2014). Rheological and microstructural characteristics of lentil starch-lentil protein composite pastes and gels. Food Hydrocoll..

[42] Earnshaw S.R., McDade C.L., Chu Y., Fleige L.E., Sievenpiper J.L. (2017). Cost-effectiveness of maintaining daily intake of oat β-glucan for coronary heart disease primary prevention. Clin. Ther..

[43] Roudi R., Mohammadi S.R., Roudbary M., Mohsenzadegan M. (2017). Lung cancer and β-glucans: Review of potential therapeutic applications. Investig. New Drugs.

[44] Pérez-Jiménez J., Torres J.L. (2011). Analysis of nonextractable phenolic compounds in foods: The current state of the art. J. Agric. Food Chem..

[45] Carcea M., Narducci V., Turfani V., Giannini V. (2017). Polyphenols in raw and cooked cereals/pseudocereals/legume pasta and couscous. Foods.

[46] Vitaglione P., Napolitano A., Fogliano V. (2008). Cereal dietary fibre: A natural functional ingredient to deliver phenolic compounds into the gut. Trends Food Sci. Technol..

[47] Milder I.E., Arts I.C., van de Putte B., Venema D.P., Hollman P.C.H. (2005). Lignan contents of Dutch plants foods: A database inscluding lariciresinol, pinoresinol, secoisolariciresinol and matairesinol. Br. J. Nutr..

[48] Adlercreutz H. (2007). Lignans and human health. Crit. Rev. Clin. Lab. Sci..

[49] During A., Debouche C., Raas T., Larondelle Y. (2012). Among plant lignans, pinoresinol has the strongest anti-inflammatory properties in human intestinal Caco-2 cells. J. Nutr..

[50] Dong S.F., Chen J.M., Zhang W., Sun S.H., Wang J., Gu J.X., Boraschi D., Qu D. (2007). Specific immune response to HBsAg is enhanced by beta-glucan oligosaccharide containing an alpha-(1-->3)-linked bond and biased towards M2/Th2. Int. Immunopharmacol..

[51] Bobadilla F., Rodriguez-Tirado C., Imarai M., Galotto M.J., Andersson R. (2013). Soluble β-1,3/1,6-glucan in seaweed from the southern hemisphere and its immunomodulatory effect. Carbohydr. Polym..

[52] Min H., Montecino-Rodriguez E., Dorshkind K. (2005). Effects of aging on early B-and T-cell development. Immunol. Rev..

[53] Riley R.L., Blomberg B.B., Frasca D. (2005). B cells, E2A, and aging. Immunol. Rev..

[54] Gollapudi S., Gupta S. (2016). Reversal of oxidative stress-induced apoptosis in T and B lymphocytes by Coenzyme Q10 (CoQ10). Am. J. Clin. Exp. Immunol..

[55] Xiao P., Huang H., Chen J., Li X. (2014). In vitro antioxidant and anti-inflammatory activities of Radix Isatidis extract and bioaccessibility of six bioactive compounds after simulated gastro-intestinal digestion. J. Ethnopharmacol..

[56] Gao S., Shu S., Wang L., Zhou J., Yuan Z. (2014). Pro-inflammatory and anti-inflammatory cytokine responses of peripheral blood mononuclear cells in apparently healthy subjects. Nan Fang Yi Ke Da Xue Xue Bao.

[57] Măluţan A.M., Drugan T., Ciortea R., Mocan-Hognogi R.F., Bucuri C., Rada M., Mihu D. (2015). Serum anti-inflammatory cytokines for the evaluation of inflammatory status in endometriosis. J. Res. Med. Sci..

[58] Teimourian M., Jafaraian Z., Hosseini S.R., Rahmani M., Bagherzadeh M., Aghamajidi A., Bijani A., Nooreddini H., Mostafazadeh A. (2016). Both immune hormone IL-10 and parathormone tend to increase in serum of old osteoporotic people independently of 1, 25 dihydroxy vitamin D3. Casp. J. Intern. Med..

[59] Langmann G.A., Perera S., Ferchak M.A., Nace D.A., Resnick N.M., Greenspan S.L. (2017). Inflammatory Markers and Frailty in Long-Term Care Residents. J. Am. Geriatr. Soc..

